# 超高效液相色谱-四极杆-飞行时间质谱法鉴定黄大茶化学成分

**DOI:** 10.3724/SP.J.1123.2023.10021

**Published:** 2024-09-08

**Authors:** Ruotong HUANG, Xuewen RONG, Xiaojie FU, Chang CHEN, Jun CHU, Na XU, Huan WU

**Affiliations:** 1.安徽中医药大学科研技术中心,安徽合肥 230038; 1. Research and Technology Center, Anhui University of Chinese Medicine, Hefei 230038, China; 2.安徽农业大学茶树生物学与资源利用国家重点实验室,安徽合肥 230036; 2. State Key Laboratory of Tea Plant Biology and Utilization, Anhui Agricultural University, Hefei 230036, China; 3.安徽省中药研究与开发重点实验室,安徽合肥 230012; 3. Anhui Province Key Laboratory of Research and Development of Chinese Medicine, Hefei 230012, China; 4.食药用菌功能活性与资源利用安徽省联合共建学科重点实验室,安徽合肥 230038; 4. Functional Activity and Resource Utilization on Edible and Medicinal Fungi Joint Laboratory of Anhui Province, Hefei 230038, China

**Keywords:** 超高效液相色谱-四极杆-飞行时间质谱, 中性丢失, 特征碎片离子, 黄大茶, 化学成分, ultra performance liquid chromatography-quadrupole time-of-flight mass spectrometry (UPLC-Q TOF/MS), neutral loss, characteristic fragment ions, large-leaf yellow tea, chemical components

## Abstract

黄大茶是中国特有的茶叶品种,具有降脂、降糖、改善代谢综合征的保健功效,但由于缺乏对黄大茶化学成分的研究,其功效性物质基础尚未被完全揭示。本研究以超高效液相色谱-四极杆-飞行时间质谱(UPLC-Q TOF/MS)作为检测工具,结合中性丢失和特征碎片离子等信息,对黄大茶的主要化学成分进行筛查和分析。选取Waters ACQUITY UPLC BEH C18色谱柱(100 mm×2.1 mm, 1.7 μm)进行分离,以0.1%甲酸水和乙腈作为流动相进行梯度洗脱,流速0.2 mL/min,进样量2 μL,柱温35 ℃。在电喷雾电离(ESI)正/负离子模式下,利用全信息串联质谱(MS^E^)技术采集黄大茶样品溶液的质谱信息。通过查阅文献构建茶叶化学成分数据库,主要包括化学名称、分子式、结构式、准分子离子、碎片离子等信息,对自建数据库中的化学成分按照骨架结构进行归类;基于UPLC-Q TOF/MS测定结果,利用对照品对主要类别化合物的质谱裂解规律进行梳理,并总结其特征碎片离子和中性丢失特征;结合化合物的保留时间、准分子离子、碎片离子等信息对化合物结构进行表征。本研究共从黄大茶中鉴定出87个化学成分,包括10个儿茶素类、32个黄酮类、16个酚酸类、12个鞣质类、6个茶黄素类以及11个其他类别化合物,其中14个成分经对照品予以验证。该方法能够全面阐明黄大茶的主要化学成分,为黄大茶功效性成分发现、品质评价提供科学依据和数据支撑。

## Abstract (en)

Large-leaf yellow tea, a slightly fermented yellow tea that is unique to China, has a stronger hypoglycemic effect than other tea varieties, such as green and black tea. Research on large-leaf yellow tea has focused on its hypoglycemic effect owing to the lack of comprehensive techniques to characterize its chemical components; thus, its development and further promotion are limited. Therefore, the development of a reliable analytical method to fully characterize the chemical components of large-leaf yellow tea is urgently required. In this study, a reliable strategy based on the data-acquisition technology of ultra performance liquid chromatography-quadrupole time-of-flight mass spectrometry (UPLC-Q TOF/MS) was established to rapidly screen and analyze the main chemical components of large-leaf yellow tea by combining the information of neutral loss groups and characteristic fragment ions. The chromatographic separation experiments were performed on a Waters ACQUITY UPLC BEH C18 column (100 mm×2.1 mm, 1.7 μm) with gradient elution using 0.1% formic acid aqueous solution and acetonitrile as the mobile phases. The flow rate was 0.2 mL/min, the sample volume was 2 μL, and the column temperature was 35 ℃. The mass spectral information of the components in a large-leaf yellow tea solution was collected using the full-information tandem MS (MS^E^) technique in positive and negative ion modes. The specific chemical components of large-leaf yellow tea was identified as follows. First, a self-established database of tea chemical components was constructed based on the literature. The mass spectral cleavage pathways of different types of compounds in large-leaf yellow tea were then sorted using reference substances, and the characteristics of the fragment ions and neutral loss groups were summarized. The precise mass-to-charge ratio of the target chemical components were then obtained based on the mass spectral information. Finally, the structures of the compounds in large-leaf yellow tea were confirmed based on their chromatographic retention times, mass spectral cleavage pathways, characteristic fragment ions, and neutral loss groups. A total of 87 chemical components, including 10 catechins, 32 flavonoids, 16 phenolic acids, 12 tannins, 6 theaflavins, and 11 compounds in other classes, were identified in large-leaf yellow tea. Representative compounds of various classes, including gallocatechin gallate, quercetin, vitexin, gallic acid, chlorogenic acid, 1,3,6-tri-*O*-galloyl-*β*-D-glucose, and theaflavin, were selected, and their characteristic fragment ions and neutral loss groups were investigated in detail to reveal the cleavage pathways of different types of compounds in large-leaf yellow tea. The UPLC-Q TOF/MS method established in this study can comprehensively identify the main chemical components of large-leaf yellow tea in a simple, highly sensitive, stable, and reliable manner. This study provides a scientific basis and data support for the discovery of functional ingredients and quality evaluation of large-leaf yellow tea.

黄大茶是中国特有的茶叶品种,主要产于安徽省霍山县、金寨县和岳西县等,其中以霍山县的黄大茶品质最优,属于轻度发酵黄茶^[1]^。独特的“闷黄”和“拉老火”高温烘焙工艺,造就了黄大茶“黄叶黄汤”的品质特征,形成独特的烘焙香、焦糖香和锅巴香,类似于咖啡/可可的烘焙香气^[2]^,并具有消垢腻、去积滞的作用。现代药理学研究表明,黄大茶具有降脂、降糖、改善代谢综合征等多种功效^[3,4]^。黄大茶可通过阻滞因高脂饮食引起的叉头转录因子O1(FOXO1)和固醇调节元件结合蛋白-1(SREBP-1)表达水平的升高,以及减少肝糖异生和增强脂肪酸分解等途径来发挥降糖和预防糖尿病的作用^[5,6]^。然而,黄大茶的功效性物质基础尚未被完全揭示,因此亟需开发出一种可靠的分析方法来全面表征黄大茶的化学成分。

超高效液相色谱-四极杆-飞行时间质谱(UPLC-Q TOF/MS)具有动态扫描范围宽、分离性能佳、分辨率和灵敏度高等特点,在天然产物的成分分离和结构表征方面具有广泛的应用^[7⇓⇓-10]^。植物提取物的成分众多、结构多样,导致质谱数据的解析工作较为繁杂。基于化合物的结构特征,从复杂的质谱数据中探寻出共性或规律性信息,将有助于减轻质谱分析工作的难度。在一定碰撞能量下,具有相同取代基或结构特征的化合物通常易发生相同质量数的中性丢失^[11]^,而核心结构相同的化合物易形成相同的特征碎片离子^[12]^;将上述两种质谱裂解规律结合,有助于实现复杂体系化合物的结构表征^[13]^。

本研究以UPLC-Q TOF/MS为检测手段,结合中性丢失和特征碎片离子等信息对黄大茶的主要化学成分进行识别,以期为黄大茶功效性物质发现、品质评价及保健功效的分子机理研究提供科学依据和数据支撑。

## 1 实验部分

### 1.1 仪器、试剂与材料

ACQUITY I Class型UPLC分离系统和Xevo G2-XS型Q TOF质谱仪检测系统(美国Waters公司); KQ-500DB型数控超声波清洗器(昆山市超声仪器有限公司); RE-3000A型旋转蒸发仪(上海亚荣生化仪器厂); Milli-Q超纯水净化系统(美国Milli-Q公司)。

黄大茶购自安徽省霍山抱儿钟秀茶业有限公司;对照品:儿茶素(批号C14501346,纯度98%)、表儿茶素(批号K9J9R66088,纯度98%)、没食子儿茶素(批号M27F114096,纯度98%)、表没食子儿茶素(批号K22N9R75650,纯度98%)、儿茶素没食子酸酯(批号S16GB159289,纯度98%)、没食子儿茶素没食子酸酯(批号A29GB147166,纯度98%)、表儿茶素没食子酸酯(批号K07J12R136470,纯度98%)、表没食子儿茶素没食子酸酯(批号C078Y39406,纯度98%)、绿原酸(批号A22GB158496,纯度98%)、槲皮素(批号O29HB199514,纯度98%)购自上海源叶生物科技有限公司;金丝桃苷(批号23030832,纯度99%)、异槲皮苷(批号22090221,纯度98%)购自上海同田生物技术股份有限公司;隐绿原酸(批号G23060508,纯度99%)购自坛墨质检标准物质中心;没食子酸(批号MUST-22112411,纯度99%)购自成都曼斯特生物科技有限公司;甲酸(LC-MS级)购自美国Sigma-Aldrich公司;甲醇(HPLC级)、乙腈(LC-MS级)购自德国Merck公司。

### 1.2 样品制备

将适量黄大茶样品粉碎后过80目筛,称取15.0 g上述样品置于500 mL锥形瓶中,按料液比为1 g∶20 mL的比例加入300 mL 75%乙醇水溶液,在360 W、60 ℃条件下超声辅助提取30 min;收集提取液,将其冷却至室温,之后对提取液进行抽滤,并将滤液置于旋转蒸发仪中,减压浓缩至1~2 mL;随后将浓缩液置于冻干机中进行冷冻干燥,24 h后即得黄大茶冻干粉(1.0 g黄大茶冻干粉相当于3.5 g黄大茶样品),低温避光保存。

### 1.3 黄大茶样品溶液和混合对照品溶液的制备

称取2.0 g黄大茶冻干粉于50 mL烧杯中,加入20 mL 70%甲醇水,超声处理(360 W)10 min,将溶液在3000 r/min下离心10 min,随后利用0.22 μm微孔滤膜将上清液过滤至进样小瓶中,即得黄大茶样品溶液。精密称取适量儿茶素、表儿茶素、没食子儿茶素、表没食子儿茶素、儿茶素没食子酸酯、没食子儿茶素没食子酸酯、表儿茶素没食子酸酯、表没食子儿茶素没食子酸酯、绿原酸、槲皮素、金丝桃苷、异槲皮苷、隐绿原酸、没食子酸对照品,分别用甲醇溶解,配制成质量浓度为1 g/L的对照品储备液;取各对照品储备液10 μL,用甲醇稀释、定容,配制成质量浓度为10 mg/L的混合对照品溶液。

### 1.4 仪器条件

#### 1.4.1 色谱条件

Waters ACQUITY UPLC BEH C18色谱柱(100 mm×2.1 mm, 1.7 μm);流动相A和流动相B分别为0.1%甲酸水和乙腈。梯度洗脱程序:0~5 min, 95%A; 5~10 min, 95%A~90%A; 10~20 min, 90%A~84%A; 20~32 min, 84%A~80%A; 32~37 min, 80%A~65%A; 37~42 min, 65%A~45%A; 42~45 min, 45%A~30%A; 45~50 min, 30%A~10%A; 50~53 min, 10%A; 53~54 min, 10%A~95%A; 54~55 min, 95%A。流速0.2 mL/min;进样量2 μL;柱温35 ℃。

#### 1.4.2 质谱条件

离子源:电喷雾电离(ESI)源,正、负离子扫描模式;将MassLynx 4.1软件(美国Waters公司)作为质谱工作站,参数设置:质谱扫描范围*m/z* 50~1200,毛细管电压3.0 kV(ESI^+^)或2.5 kV(ESI^-^),锥孔电压40 V,离子源温度120 ℃,去溶剂化温度350 ℃,溶剂气流量600 L/h。全信息串联质谱(MS^E^)的低碰撞能量设置为6 V,高碰撞能量设置为50~80 V。在进样分析前,利用调谐液校正Q TOF/MS的质量轴;在数据采集过程中,使用亮氨酸脑啡肽进行实时校准。在ESI^+^和ESI^-^模式下分别选择*m/z* 556.2771 [M+H]^+^和*m/z* 554.2615 [M-H]^-^作为实时矫正质量数。

### 1.5 数据分析方法

本研究以“茶”“化学成分”“tea”“chemical components”为关键词,分别在中国知网、PubMed、Chemical Book等数据库中进行检索,整理检索所得茶叶化学成分信息,包括化学名称、分子式、结构式、准分子离子、碎片离子等,制作成电子表格;对数据库中的化学成分按照核心结构进行归类,同时绘制其结构式,并保存为MOL文件,即得茶叶化学成分的自建数据库。通过对照品来获得儿茶素类、黄酮类、酚酸类成分的质谱裂解特征,收集其特征碎片离子和中性丢失信息;同时,结合自建数据库及参考文献来获取其他类别成分的质谱信息。利用UPLC-Q TOF/MS技术获得质谱数据,并在黄大茶样品的总离子流色谱图中对各化合物进行峰提取,实现黄大茶样品溶液中化学成分的初筛,然后依据特征碎片离子、中性丢失及部分对照品等信息对化学成分结构进行表征。

## 2 结果与讨论

### 2.1 提取条件的优化

本研究分别考察了不同提取溶剂(超纯水、乙醇、乙酸乙酯、石油醚)和不同提取方式(超声辅助提取、回流提取)对黄大茶化学成分提取效果的影响,并根据样品总离子流色谱图中的峰个数、总峰面积等指标来确定样品提取条件。实验结果表明,将乙醇作为提取溶剂,经超声辅助提取后,黄大茶样品溶液总离子流色谱图的总峰面积最大且峰数目最多,化合物提取效果最佳。

采用单因素变量法,继续参照上述评价指标,考察不同体积分数(25%、50%、75%、100%)的乙醇水、不同料液比(1 g∶10 mL、1 g∶20 mL、1 g∶30 mL、1 g∶40 mL)、不同提取温度(50、60、70、80 ℃)及不同提取时间(15、30、45、60 min)对黄大茶化学成分提取效果的影响。结果表明,以75%乙醇水为提取溶剂时,色谱峰的个数较多且信号强度最佳;在不同料液比样品的总离子流色谱图中,色谱峰总数无明显差异,但色谱图的总峰面积随溶剂比例的增大呈现先升高、后降低的趋势,当料液比为1 g∶20 mL时,总峰面积达到最大值;当提取温度为60 ℃时,总峰面积达到最大值,继续升高温度,总峰面积逐渐减小;随着提取时间的延长,色谱峰的总面积先增大、后减小,在30 min时达到最大。综上,选择75%乙醇水为提取溶剂,料液比为1 g∶20 mL,提取温度为60 ℃,提取时间为30 min,提取方式为超声辅助提取。

### 2.2 UPLC-Q TOF/MS条件的优化

本研究考察了不同有机相(乙腈、甲醇)与不同水相(0.1%甲酸水、0.5%乙酸水)作为流动相时,对黄大茶中各化合物的分离情况。结果显示,将乙腈和0.1%甲酸水作为流动相时,分离基线更为平稳,色谱峰形良好,因此选择乙腈和0.1%甲酸水作为流动相。黄大茶中化学成分众多,当洗脱时间较短时,极性相近化合物的色谱峰易重叠,裂解产生的碎片离子峰会互相干扰,导致结构表征的难度增大。通过调整梯度洗脱程序,改善分离情况,可获取更为准确的碎片离子信息,因此对梯度洗脱程序进行优化,优化结果见1.4.1节。结果表明,在优化的梯度洗脱程序下,黄大茶中化学成分的整体色谱峰形较好,且绝大多数组分能够达到理想的分离效果。此外,本研究还对比了黄大茶样品在不同碰撞能量(30~50、40~60、50~80 V)下的二级质谱图,结果显示,当碰撞能量为50~80 V时,多数化合物的碎片离子信息最为丰富,因此选择在50~80 V碰撞能量下诱导化合物解离。

### 2.3 黄大茶化学成分的分析

在优化的实验条件下,黄大茶样品溶液在ESI^-^和ESI^+^模式下的总离子流色谱图如图1a和图1b所示,混合对照品溶液在ESI^-^模式下的总离子流色谱图见图1c。本研究共识别出黄大茶中的87个化学成分,包括10个儿茶素类、32个黄酮类、16个酚酸类、6个茶黄素类、12个鞣质类以及11个其他类别化合物,相应的分子式、化合物名称、特征碎片离子及中性丢失等信息列于表1,同时利用对照品对其中14个化合物予以验证。以70%甲醇水为空白样品溶液,其在ESI^-^/ESI^+^模式下的总离子流色谱图见图2。

**图1:**
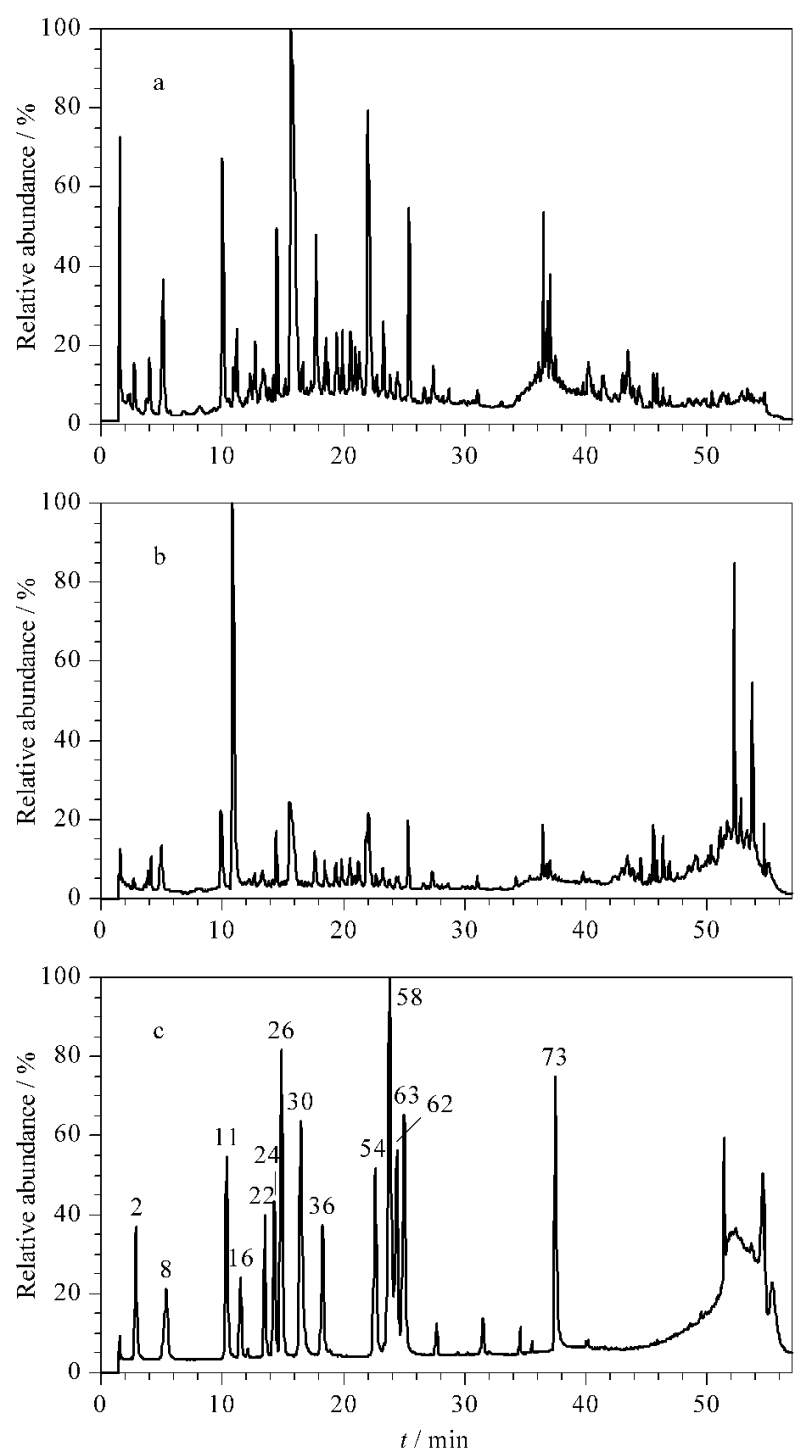
黄大茶样品溶液在(a)ESI^-^和(b)ESI^+^模式下的 总离子流色谱图;(c)混合对照品溶液在ESI^-^模式下的总离子流色谱图

**表1:** 黄大茶中87种化学成分的LC-MS分析与鉴定

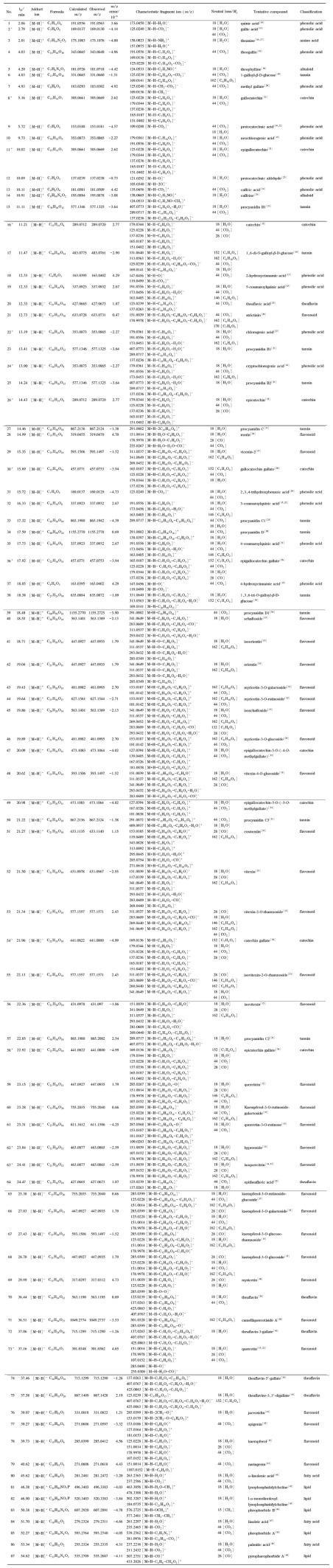

m/z error= ( observed m/z- calculated m/ z)/ calculated m/z; * confirmed by reference substance, and the corresponding references were used to determine the fragment ions.

**图2:**
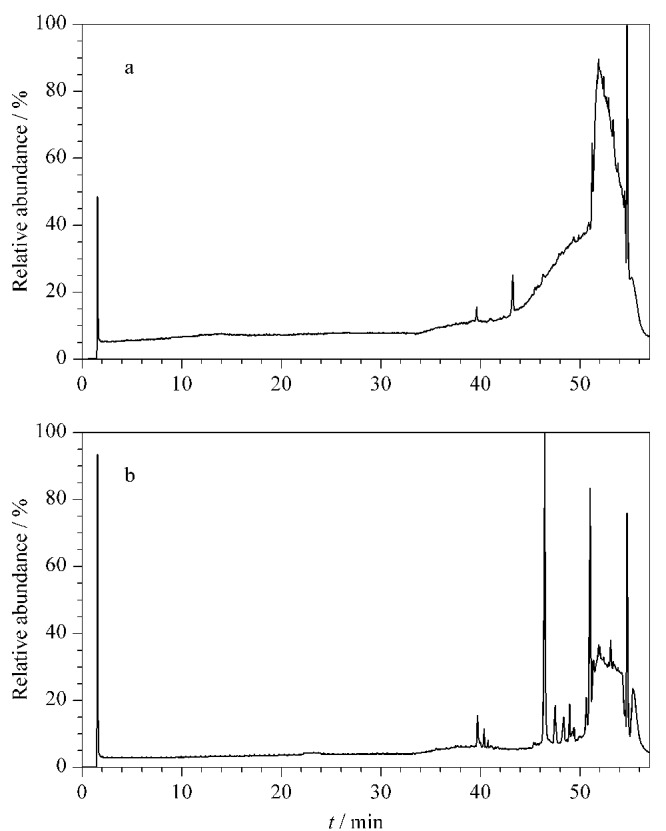
空白样品溶液在(a)ESI^-^和(b)ESI^+^模式下的总离子流色谱图

对黄大茶样品中不同种类化合物的特征碎片离子和中性丢失规律进行总结,详细如下:(1)儿茶素类化合物可产生*m/z* 179 [C_9_H_8_O_4_]^-^、*m/z* 165 [C_8_H_6_O_4_]^-^、*m/z* 151 [C_8_H_8_O_3_]^-^、*m/z* 137 [C_7_H_6_O_3_]^-^和*m/z* 125 [C_6_H_6_O_3_]^-^特征碎片离子,还可以发生44 [CO_2_]、28 [CO]和18 [H_2_O]的中性丢失。(2)黄大茶中黄酮类成分的特征碎片离子除*m/z* 151 [C_7_H_4_O_3_]^-^和*m/z* 125 [C_6_H_6_O_3_]^-^等典型碎片外,还有*m/z* 285 [C_15_H_10_O_6_]^-^、*m/z* 255 [C_14_H_8_O_5_]^-^、*m/z* 269 [C_15_H_10_O_5_]^-^、*m/z* 341 [C_18_H_14_O_7_]^-^和*m/z* 311 [C_17_H_12_O_6_]^-^等其他碎片。多数黄酮类化合物可发生44 [CO_2_]、28 [CO]和18 [H_2_O]等小分子的中性丢失;具有葡萄糖基、半乳糖基、鼠李糖基、阿拉伯糖基等取代基的黄酮类化合物还可相应发生162[C_6_H_10_O_5_]、162[C_6_H_10_O_5_]、146[C_6_H_10_O_4_]和132[C_5_H_8_O_4_]的中性丢失。(3)酚酸类化合物易产生*m/z* 179 [C_9_H_8_O_4_]^-^、*m/z* 191 [C_7_H_12_O_6_]^-^和*m/z* 125 [C_6_H_6_O_3_]^-^特征碎片离子,易发生44 [CO_2_]和18 [H_2_O]的中性丢失。(4)黄大茶中的水解鞣质类化合物易产生*m/z* 313 [C_13_H_14_O_9_]^-^、*m/z* 331 [C_13_H_16_O_10_]^-^特征碎片离子及152 [C_7_H_4_O_4_]、44 [CO_2_]和18 [H_2_O]的中性丢失;缩合鞣质类化合物可产生*m/z* 289 [C_15_H_14_O_6_]^-^、*m/z* 137 [C_7_H_6_O_3_]^-^特征碎片离子及44 [CO_2_]和18 [H_2_O]的中性丢失。(5)黄大茶中的茶黄素类化合物具有与儿茶素类化合物相似的裂解规律,可产生*m/z* 425 [C_22_H_18_O_9_]^-^、*m/z* 407 [C_22_H_16_O_8_]^-^、*m/z* 125 [C_6_H_6_O_3_]^-^、*m/z* 137 [C_7_H_6_O_3_]^-^特征碎片离子及18 [H_2_O]的中性丢失。

### 2.4 黄大茶中各类化学成分的分析与鉴定

#### 2.4.1 儿茶素类

儿茶素类化合物是茶叶中含量占比(约为60%~80%)最多的多酚化合物^[50]^,多数儿茶素类化合物具有苯并二氢吡喃的基本结构。根据化合物结构中C环C-3位置上的羟基是否被没食子酸(gallic acid, Ga)酯化,可将儿茶素类化合物分为酯型儿茶素或简单儿茶素。

本研究通过儿茶素对照品所获得的儿茶素类化合物质谱裂解规律如图3所示,因其C环C-3位置上存在羟基或Ga等取代基,在高碰撞能量下,儿茶素类化合物易丢失18 [H_2_O]或170 [Ga]等中性分子;酯型儿茶素类化合物易丢失没食子酰基,形成高丰度的骨架碎片离子,之后的裂解途径与简单儿茶素一致,主要包括C环的逆狄尔斯-阿尔德(retro-Diels-Alder reaction, RDA)裂解与B环的丢失以及44 [CO_2_]、18 [H_2_O]、28 [CO]等小分子的中性丢失。结合上述质谱裂解特征和对照品信息,共鉴定出黄大茶中的10个儿茶素类化合物,结果见表1。

**图3:**
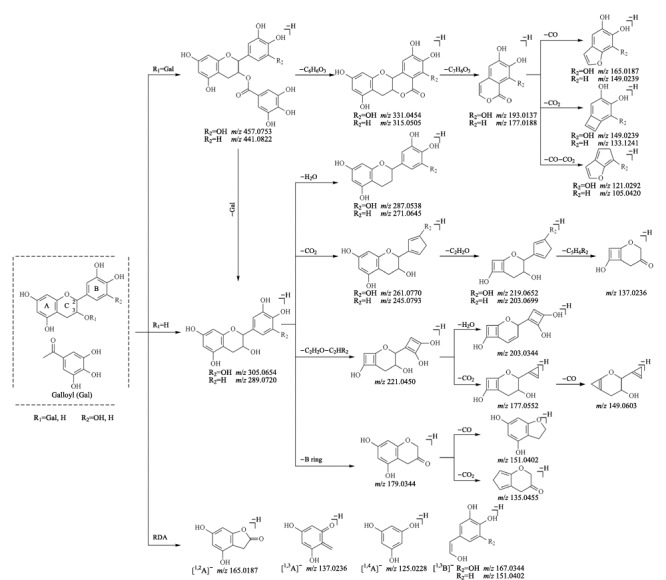
儿茶素类化合物的裂解途径

30号化合物在ESI^-^模式下的保留时间为15.69 min,准分子离子峰为*m/z* 457.0753,分子式为C_22_H_18_O_11_。在高碰撞能量作用下,该化合物丢失没食子酰基,生成骨架碎片离子(*m/z* 305.0654 [M-H-C_7_H_4_O_4_]^-^);之后,该骨架碎片离子发生C环1,2、1,3、1,4键的断裂以及B环的丢失,分别产生4个特征碎片离子,即*m/z* 165.0187 [M-H-C_7_H_4_O_4_-C_7_H_8_O_3_]^-^、*m/z* 137.0236 [M-H-C_7_H_4_O_4_-C_8_H_8_O_4_]^-^、*m/z* 125.0228 [M-H-C_7_H_4_O_4_-C_9_H_8_O_4_]^-^和*m/z* 179.0344 [M-H-C_7_H_4_O_4_-C_6_H_6_O_3_]^-^;上述骨架碎片离子还可以通过丢失一个中性分子CO_2_,产生碎片离子*m/z* 261.0770。此外,30号化合物丢失一分子Ga,可产生碎片离子*m/z* 287.0538,同时该碎片离子还可由上述骨架碎片离子丢失一分子H_2_O产生。结合以上裂解规律和对照品信息,判定30号化合物为没食子儿茶素没食子酸酯。其余9个儿茶素类化合物均按照类似方法进行鉴定。

#### 2.4.2 黄酮类

黄酮类化合物是一类以2-苯基色原酮为基本骨架的天然次级代谢产物^[51]^,在植物中少数黄酮类化合物以游离苷元形式存在,多数以与糖结合形成苷类的形式存在,包括黄酮氧苷类与黄酮碳苷类。通过对照品及文献[14,15,26]可获得黄酮苷元及黄酮氧苷类化合物的质谱裂解规律,具体如下:在质谱碰撞诱导解离中,黄酮苷类化合物结构中的糖基(如鼠李糖、葡萄糖、半乳糖等)在发生中性丢失后可产生相应的黄酮苷元,黄酮苷元中的C环经RDA裂解可产生[^1,2^A]^-^、[^1,3^A]^-^和[^1,4^B]^-^等系列特征碎片离子,同时黄酮苷元还可以发生44 [CO_2_]、28 [CO]、18 [H_2_O]等小分子的中性丢失;黄酮碳苷类化合物除发生上述质谱裂解行为外,还可发生糖环的开环裂解,产生与黄酮氧苷区分度明显的特征性碎片^[52]^。结合上述裂解特征及对照品质谱信息,共鉴定出黄大茶中的32个黄酮类化合物。

73号化合物在ESI^-^离子模式下的保留时间为37.16 min,准分子离子峰为*m/z* 301.0362,分子式为C_15_H_10_O_7_。该化合物C环中的0,4、1,2和1,3键发生RDA裂解后,分别形成特征碎片离子*m/z* 107.0152 [M-H-C_9_H_6_O_5_]^-^、*m/z* 178.9978 [M-H-C_7_H_6_O_2_]^-^和*m/z* 151.0014 [M-H-C_8_H_6_O_3_]^-^; 73号化合物丢失一个O原子后可产生特征碎片离子*m/z* 285.0400,丢失一分子H_2_O后可产生碎片离子*m/z* 283.0265,碎片离子*m/z* 283.0265继续丢失一分子CO后产生碎片离子*m/z* 255.0300; 73号化合物丢失一分子CO_2_产生碎片离子*m/z* 257.0433后,继续丢失一分子CO产生*m/z* 229.0483碎片;73号化合物丢失一分子CH_2_O可产生碎片离子*m/z* 271.0241,继续丢失一分子CO产生碎片离子*m/z* 243.0274。综上,判定73号化合物为槲皮素,其二级质谱图及裂解途径分别见图4a和图4b。

**图4:**
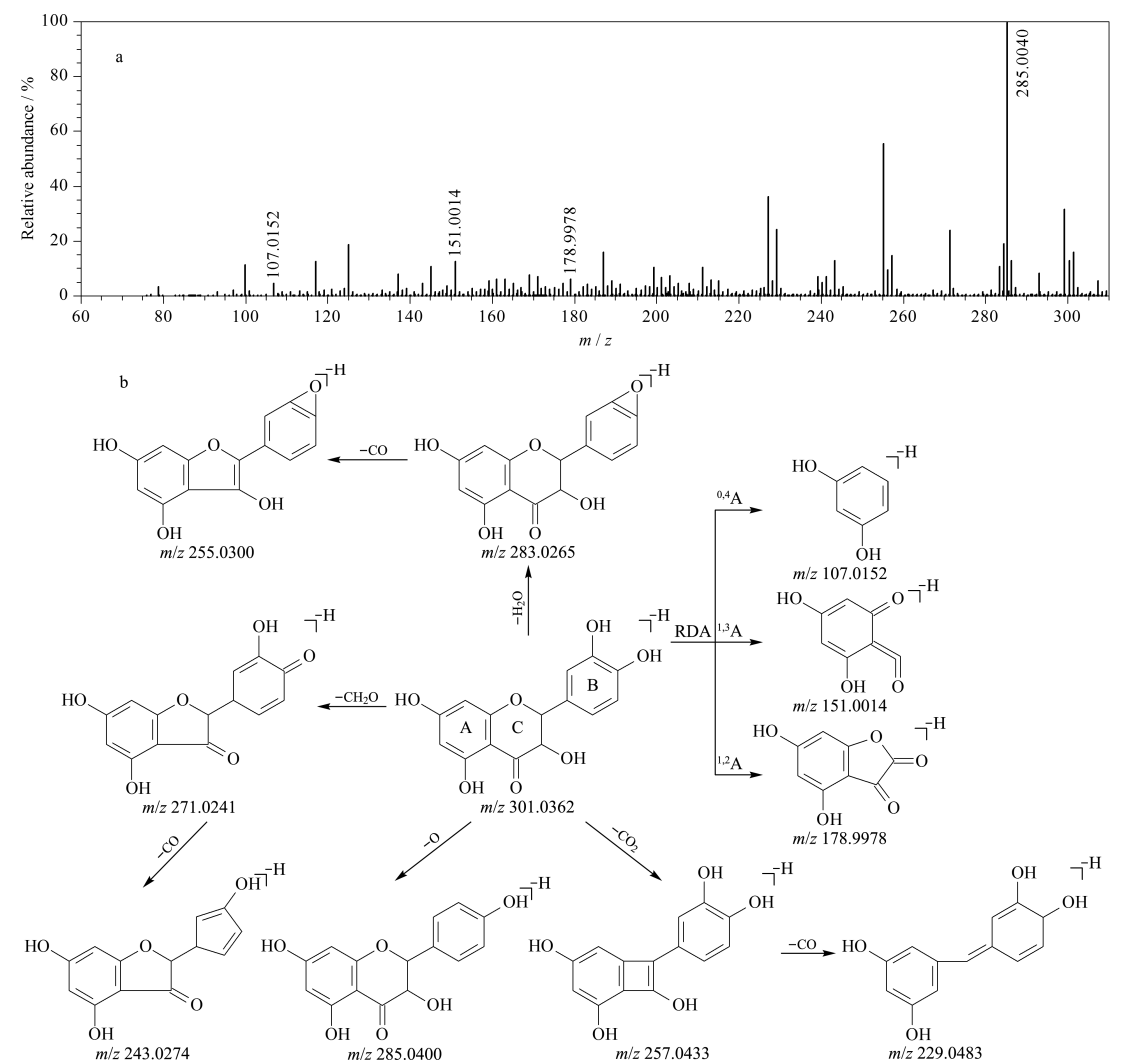
槲皮素在ESI^-^模式下的(a)二级质谱图和(b)裂解途径

52号化合物在ESI^-^模式下的保留时间为21.30 min,准分子离子峰为*m/z* 431.0967,分子式为C_21_H_20_O_10_。该化合物丢失葡萄糖基可产生特征碎片离子*m/z* 269.0440 [M-H-C_6_H_10_O_5_]^-^,该碎片C环的1,3键随之发生RDA裂解,分别形成特征碎片离子*m/z* 151.0039 [M-H-C_6_H_10_O_5_-C_8_H_6_O]^-^和*m/z* 117.0339 [M-H-C_6_H_10_O_5_-C_7_H_4_O_4_]^-^;该化合物也可发生葡萄糖基内部的开环裂解,从而产生黄酮碳苷的特征碎片离子(*m/z* 341.0649 [M-H-C_3_H_6_O_3_]^-^和*m/z* 311.0537 [M-H-C_4_H_8_O_4_]^-^),其中*m/z* 311.0537对应两种*m/z*相同、但双键位置不同的碎片离子,这两种碎片离子分别丢失一分子H_2_O和一分子CO后产生特征碎片离子*m/z* 293.0432和*m/z* 283.0609。此外,52号化合物丢失一分子H_2_O可产生碎片离子*m/z* 413.0872。结合文献[53],推测52号化合物为牡荆素,其二级质谱图及裂解途径分别见图5a和图5b。其余30个黄酮类化合物均按照类似方法进行识别。

**图5:**
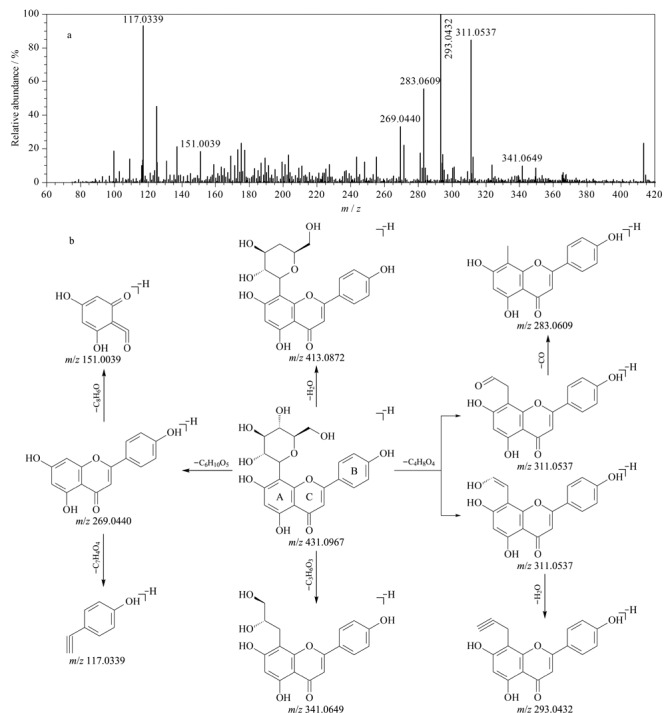
牡荆素在ESI^-^模式下的(a)二级质谱图和(b)裂解途径

#### 2.4.3 酚酸类

酚酸是茶叶中普遍存在且具有酚羟基、羧基等酸性基团的次生代谢产物^[54]^。在质谱高碰撞能量下,酚酸类化合物极易发生18 [H_2_O]和44 [CO_2_]等小分子的中性丢失。茶叶中的酚酸类化学成分主要包括咖啡酸类、奎尼酸类和没食子酸类^[55]^,在质谱高碰撞能量下,三者分别容易产生特征碎片离子*m/z* 179.0361 [C_9_H_8_O_4_-H]^-^、*m/z* 191.0556 [C_7_H_12_O_6_-H]^-^和*m/z* 125.0240 [C_6_H_6_O_3_-H]^-^。基于以上裂解特征,结合对照品及文献[14,15,23,27],共鉴定出黄大茶中的16个酚酸类化合物。

2号化合物在ESI^-^模式下的保留时间为2.79 min,准分子离子峰为*m/z* 169.0130,分子式为C_7_H_6_O_5_。该化合物的碎片离子主要通过丢失CO_2_产生,如特征碎片离子*m/z* 125.0240 [M-H-CO_2_]^-^,结合对照品,判定该化合物为Ga。22号化合物在ESI^-^模式下的保留时间为13.19 min,准分子离子峰为*m/z* 353.0865,分子式为C_16_H_18_O_9_。在质谱中可观察到该化合物的特征碎片离子*m/z* 179.0361 [M-H-C_7_H_10_O_5_]^-^、*m/z* 191.0556 [M-H-C_9_H_6_O_3_]^-^,其中碎片离子*m/z* 191.0556继续丢失一分子H_2_O或CO_2_,分别形成特征碎片离子*m/z* 173.0453 [M-H-C_9_H_6_O_3_-H_2_O]^-^和碎片离子*m/z* 147.0657 [M-H-C_9_H_6_O_3_-CO_2_]^-^。上述裂解途径与文献[56]报道一致,结合对照品信息,判定22号化合物为绿原酸。其他14个酚酸类化合物均按照类似方法进行识别。

#### 2.4.4 鞣质类

本研究共鉴定出黄大茶中的12个鞣质类化合物,包括9个缩合鞣质及3个水解鞣质化合物。缩合鞣质是以儿茶素为基本单元缩合而成的一类化合物,其具有与儿茶素类化合物相似的质谱裂解特征。黄大茶中的水解鞣质均为没食子鞣质类化合物,该类化合物是一种以葡萄糖为核心且通过酯键与Ga连接的可水解鞣质,在质谱碰撞能量作用下,该类化合物可发生18 [H_2_O]和44 [CO_2_]等小分子的中性丢失,但其特征碎片离子的生成主要归因于没食子酰基的规律性丢失。

以38号化合物为例,其在ESI^-^模式下的准分子离子峰为*m/z* 635.0872,分子式为C_27_H_24_O_18_。该化合物连续丢失两分子没食子酰基,可产生特征碎片离子*m/z* 331.0640 [M-H-C_7_H_4_O_4_-C_7_H_4_O_4_]^-^,该碎片离子继续丢失一分子H_2_O,产生特征碎片离子*m/z* 313.0563 [M-H-C_7_H_4_O_4_-C_7_H_4_O_4_-H_2_O]^-^; 38号化合物在丢失一分子Ga后可产生*m/z* 169.0141 [M-H-C_20_H_18_O_13_]^-^碎片离子。以上裂解特征与文献[57]描述一致,因此推测该化合物为三没食子酰葡萄糖。其余11个鞣质类化合物均按照类似方法进行鉴定。

#### 2.4.5 茶黄素类

茶黄素是儿茶素和没食子儿茶素通过配对氧化形成的一类具有水溶性和多羟基取代的苯并卓酚酮类化合物^[58]^,因此茶黄素类化合物具有与儿茶素类化合物近似的裂解特征。本研究共鉴定出黄大茶中的6个茶黄素类化合物。以70号化合物为例,其在ESI^-^模式下的准分子离子峰为*m/z* 563.1195,分子式为C_29_H_24_O_12_。该化合物C环位置上1,3、1,4键发生RDA裂解后,可产生特征碎片离子*m/z* 425.0863[M-H-C_7_H_6_O_3_]^-^、137.0263 [M-H-C_22_H_18_O_9_]^-^、125.0239 [M-H-C_23_H_18_O_9_]^-^等,其中碎片离子*m/z* 425.0863丢失一分子H_2_O后形成特征碎片离子*m/z* 407.0767 [M-H-C_7_H_6_O_3_-H_2_O]^-^。此外,该化合物还可直接丢失一分子H_2_O,形成碎片离子*m/z* 545.1051。结合以上裂解特征及文献[16],推测70号化合物为茶黄素。其余5个茶黄素类化合物均按照类似方法进行鉴定。

#### 2.4.6 其他类

本研究共鉴定出11个其他类别的化学成分,主要包括脂肪酸类、生物碱类、氨基酸类、脂类等。以84号化合物为例,其在ESI^-^模式下的准分子离子峰为*m/z* 279.2311,分子式为C_18_H_32_O_2_。该化合物可通过丢失一分子H_2_O形成特征碎片离子*m/z* 261.2207 [M-H-H_2_O]^-^,也可以通过丢失一分子CO_2_形成特征碎片离子*m/z* 235.2465 [M-H-CO_2_]^-^。以上裂解特征与文献[47]描述一致,因此推测84号化合物为脂肪酸类中的亚油酸。

## 3 结论

本研究构建了黄大茶化学成分的自建数据库,通过与对照品进行比对,获得了儿茶素类、黄酮类、酚酸类化合物的质谱裂解特征,收集到相关特征碎片离子和中性丢失信息。同时根据自建数据库和参考文献,总结了鞣质类、茶黄素类、生物碱类、脂肪酸类等化合物的质谱裂解特征。本研究基于UPLC-Q TOF/MS技术,结合特征碎片离子和中性丢失信息,从黄大茶样品中共识别出87个化学成分,其中10个儿茶素类、32个黄酮类、16个酚酸类、12个鞣质类、6个茶黄素类以及11个其他类别化学成分。本研究所建立方法能够用于黄大茶中主要化学成分的分析与鉴定,并为黄大茶功效性成分发现、品质评价提供科学依据和数据支撑。
